# Paediatric Index of Mortality 3 in Sweden: Validation and Recalibration to Improve Mortality Prediction in Paediatric Intensive Care

**DOI:** 10.1111/apa.70603

**Published:** 2026-05-20

**Authors:** Lars Engerström, Shanay Daham, Staffan Eksborg, Tova Hannegård Hamrin, Jonas Berner

**Affiliations:** ^1^ Department of Anaesthesiology and Intensive Care and Department of Biomedical and Clinical Sciences Linköping University Norrköping Sweden; ^2^ Department of Thoracic and Vascular Surgery and Department of Medical and Health Sciences Linköping University Linköping Sweden; ^3^ Department of Paediatric Perioperative Medicine and Intensive Care Karolinska University Hospital Stockholm Sweden; ^4^ Department of Physiology and Pharmacology Karolinska Institutet Stockholm Sweden; ^5^ Department of Women's and Children's Health, Childhood Cancer Research Unit Karolinska Institutet Stockholm Sweden

**Keywords:** intensive care, mortality, Paediatric Index of Mortality 3, paediatric intensive care unit, validation

## Abstract

**Aim:**

In Sweden, critically ill children are admitted to one of four dedicated paediatric intensive care units (PICUs) or general intensive care units (ICUs), reflecting a partly centralised system. The Paediatric Index of Mortality 3 (PIM3) is a widely used risk stratification tool for predicting mortality in critically ill children. This study aimed to validate PIM3 in a national Swedish cohort, recalibrate the model where indicated and compare outcomes between PICUs and ICUs.

**Methods:**

This retrospective cohort study used prospectively collected data from the Swedish Intensive Care Registry of admissions reported between 1 January 2016 and 30 September 2022. Discrimination and calibration were assessed, and recalibration was performed using logistic regression.

**Results:**

19 183 admissions were included, 10 714 males and 8469 females. Prior to recalibration, PIM3 overestimated mortality in both settings, with standardised mortality rates (SMRs) of 0.72 (95% Confidence Interval (CI) 0.64–0.80) in PICUs and 0.52 (95% CI 0.42–0.63) in ICUs. Following recalibration, SMR was 0.92 (95% CI 0.70–1.20) and 0.96 (95% CI 0.59–1.49) in PICUs and ICUs, respectively.

**Conclusion:**

PIM3 demonstrated excellent discrimination but systematically overestimated mortality in the Swedish context. Recalibration corrected this bias and attenuated outcome differences between PICU and ICU settings.

## Summary

– Risk‐adjusted outcome comparisons are essential where critically ill children are treated across dedicated and general intensive care units, yet the Paediatric Index of Mortality 3 has not been validated in Sweden.
– Across over 19 000 admissions, the model showed excellent discrimination but overestimated mortality; recalibration corrected this bias and attenuated outcome differences.
– Interpretation of outcome differences between unit types should be careful.

**Abbreviations**
AUC‐ROC: area under the receiver operating characteristic curve
CI: confidence interval
ECMO: extra corporeal membrane oxygenation
FiO_2_
: fraction of inspired oxygen
ICU: intensive care unit
IQR: interquartile range
PICU: paediatric intensive care unit
PIM: Paediatric Index of Mortality
ROC: receiver operating characteristic
SMR: standardised mortality rate

## 1 Introduction

In the past decades, paediatric intensive care has been increasingly centralised to regional tertiary paediatric intensive care units (PICUs). This centralisation aims to consolidate expertise, enhance resource allocation and ultimately improve patient outcomes [1, 2, 3]. Sweden is a geographically large country with a relatively sparse population, served by 84 intensive care units of varying specialisation. Only four are dedicated PICUs, located in Lund, Gothenburg, Uppsala and Stockholm. Critically ill children requiring prolonged care are generally transferred to one of these tertiary centres from general units elsewhere in the country. However, studies have reported conflicting findings regarding whether PICUs reduce risk‐adjusted mortality compared to general intensive care units (ICUs) [4, 5]. A national Swedish study examined paediatric admissions across all PICUs and ICUs between 2008 and 2012 and reported a crude ICU mortality rate of 2.0% in PICUs compared to 1.1% in ICUs. Among children under 3 years of age, however, mortality was notably higher in ICUs (4.9%) than in PICUs (2.7%) [4].

A national guideline introduced in 2015 recommended that children younger than 3 years be admitted to dedicated PICUs [6]. By 2022, approximately 50% of ICU admissions for patients up to 15 years of age were taking place in one of Sweden's four PICUs [7]. Despite this, risk‐adjusted outcome comparisons between PICUs and ICUs have not been evaluated since the implementation of the guideline.

Clinical benchmarking is essential for assessing the quality of intensive care and comparing performance across units. Risk adjustment is a prerequisite for valid comparison, accounting for differences in baseline characteristics and illness severity between patient groups. The Paediatric Index of Mortality 3 (PIM3) is the most widely used scoring tool in Scandinavian PICUs and ICUs. It calculates the predicted mortality risk using physiological and clinical parameters recorded from initial patient contact to 1 h after the ICU admission, forming the basis for risk‐adjusted standardised mortality rates (SMRs).

The PIM was developed using logistic regression on large PICU and ICU admission datasets from Australia, New Zealand, Ireland and the United Kingdom. The PIM3 represents the third iteration of the model, each revision reflecting advances in paediatric critical care [8, 9, 10]. Internal validation studies from these regions have demonstrated strong model performance. External validation studies, however, have yielded mixed results, highlighting the need for further assessment in diverse healthcare settings [11, 12]. Variability in patient demographics and disease burden may influence model accuracy, making external validation essential for reliable mortality prediction [12, 13]. In the United Kingdom and Ireland, the Paediatric Intensive Care Audit Network routinely reports PIM3 outcomes. The most recent report (2021–2023) recorded a risk‐adjusted SMR of 0.96, indicating performance close to expected outcomes [14]. For a mortality prediction tool to be meaningful, it must demonstrate both discrimination—the ability to distinguish survivors from non‐survivors, and calibration—the alignment of predicted and observed mortality. While early studies validating PIM3 indicated good discrimination, robust calibration assessments, such as through calibration belt plots, remain scarce.

This study aimed to evaluate PIM3 performance in the Swedish paediatric population, recalibrating the model to national data where indicated. Validity for predicting mortality was then assessed by comparing children admitted to both PICUs and ICUs.

## 2 Methods

### 2.1 Study Design and Setting

We conducted a retrospective observational study using data from the Swedish Intensive Care Registry. This national clinical database includes PICU and ICU admissions across Sweden [15]. The study cohort consisted of children aged up to 15 years admitted to any participating unit between 1 January 2016 and 30 September 2022.

### 2.2 Ethics

The study was approved by the Swedish Ethical Review Authority (No. 2020‐00368 0512). A waiver of informed consent was obtained due to the use of de‐identified registry data.

### 2.3 Study Population

**FIGURE 1:**
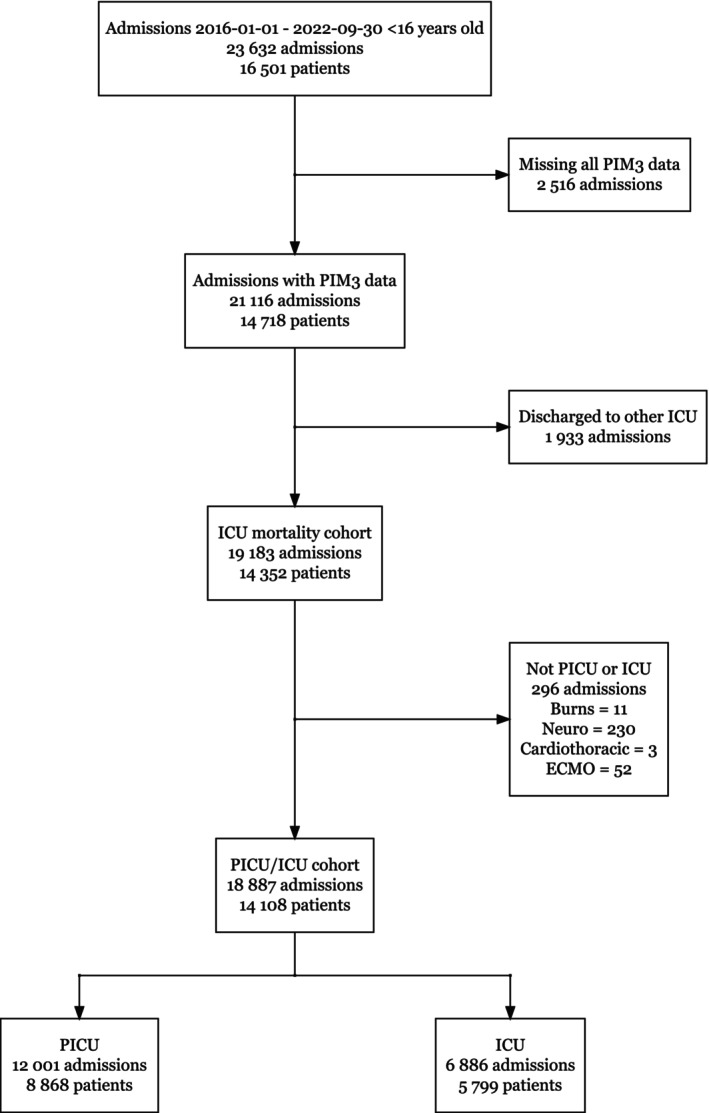
Flowchart showing inclusion and exclusion of paediatric intensive care admissions in Sweden between 1 January 2016 and 30 September 2022. Admissions were excluded if missing all PIM3 data or if transferred to another ICU. The cohort used for model development and validation comprised 19 183 paediatric ICU admissions to paediatric intensive care units (PICU) and intensive care units. For comparison of PICU and ICU, there were additional exclusions of specialised units (burns, neurosurgical, cardiothoracic and ECMO units). Numbers of admissions and unique patients are indicated at each step.

We included paediatric admissions with at least partially complete data for variables required to calculate the PIM3 score. Admissions were excluded if they lacked mortality status at discharge or if they were transferred to another ICU. As ICU mortality was the primary outcome of interest, care cannot be considered complete when a patient is transferred, in accordance with the original PIM3 methodology [10]. Patients were stratified by unit type, PICU versus ICU, for subgroup analyses and patients admitted to specialised units, such as burns units, were then excluded (Figure 1).

### 2.4 Variables and Definitions

The primary outcome was PICU and ICU mortality. The primary predictor of mortality was the PIM3 score, calculated based on standard variables available in the Swedish Intensive Care Registry, including physiological and diagnostic information at PICU and ICU admission.

### 2.5 Statistical Analysis

Patient characteristics were summarised with medians or proportions as appropriate. Missing data were substituted with predefined normal values, in accordance with the original PIM3 methodology. This approach assumes data are not missing at random, with missing measurements typically reflecting a clinical judgement that significant abnormality is unlikely [10].

Model performance was assessed in terms of discrimination and calibration. Discrimination was quantified using the area under the receiver operating characteristic curve (AUC‐ROC) [16], with confidence intervals (CI) calculated using DeLong's method [17]. Calibration was assessed using standardised mortality ratios (SMRs), with 95% CIs calculated using Byar's approximation [18], and calibration belt plots to visualise estimated versus observed mortality [19, 20]. The Hosmer‐Lemeshow goodness‐of‐fit test with 10 equally sized groups was used, which, despite known sensitivity to sample size, remains a useful tool for comparing models within the same dataset [21].

The original and recalibrated models were compared by examining differences in discrimination and calibration across unit types in PICU and ICU. Statistical significance was defined as a two‐sided *p*‐value < 0.05.

Recalibration was performed using logistic regression, applying both overall correction coefficients and full recalibration of individual coefficients. The dataset was randomly split into 80% for model development and 20% for validation. For the simple recalibration, the PIM3 score was used as the sole predictor of ICU mortality. For the full recalibration, elastic net regularisation was applied to shrink coefficients toward zero to reduce the risk of overfitting [22]. All analyses were conducted using R version 4.3.1 (R Foundation for Statistical Computing, Vienna, Austria).

## 3 Results

Between 1 January 2016 and 30 September 2022, 23 632 paediatric admissions involving 16 501 patients up to 15 years were reported to the Swedish Intensive Care Registry. Registry reporting is voluntary, and six ICUs did not contribute data in 2016. These units accounted for approximately 1% of paediatric ICU admissions in Sweden in 2021, with negligible impact on data completeness. All Swedish ICUs reported from 2021 onwards.

In the Swedish registry, variables describing the cause of admission are mandatory when PIM3 is recorded. However, the PIM3 was not registered for 2516 admissions, primarily because 21 ICUs did not use the PIM3 during the study period. In addition, 1933 admissions that were discharged to another ICU were excluded in accordance with the original PIM3 methodology, as ICU mortality is the outcome of interest and ICU care cannot be considered complete when a patient is transferred.

The remaining 19 183 admissions, comprising 8469 female and 10 714 male admissions, were used for recalibration of the model. For comparison of outcomes between PICU and ICU, an additional 296 admissions to specialist ICUs were excluded (Figure 1). Baseline characteristics for all admissions are summarised in Table 1 and treatments received are detailed in Table 2.

**TABLE 1:** Baseline characteristics of admissions in all the ICU‐mortality cohort.

Variable	Overall	Deceased	Survived	*p*	Missing
No.	19 183	452	18 731		
Age, years (median [IQR])	2.0 [0.0 to 9.0]	1.0 [0.0 to 8.0]	2.0 [0.0 to 9.0]	0.023	0.0
Gender male (%)	10 714 (55.9)	237 (52.4)	10 477 (55.9)	0.152	0.0
ICU LOS, h (median [IQR])	23.2 [14.6 to 59.6]	66.7 [24.2 to 157.1]	23.0 [14.5 to 56.0]	< 0.001	0.0
Unplanned admission = No. (%)	6276 (32.7)	32 (7.1)	6244 (33.3)	< 0.001	0.0
Very high risk admission (%)	879 (4.6)	212 (46.9)	667 (3.6)	< 0.001	0.0
High risk admission (%)	954 (5.0)	59 (13.1)	895 (4.8)	< 0.001	0.0
Low risk admission (%)	3394 (17.7)	3 (0.7)	3391 (18.1)	< 0.001	0.0
Postoperative care (%)	7162 (37.3)	57 (12.6)	7105 (37.9)	< 0.001	0.0
Cardiac surgery with ECC (%)	2457 (12.8)	19 (4.2)	2438 (13.0)		
Cardiac surgery without ECC (%)	393 (2.0)	2 (0.4)	391 (2.1)		
Non‐cardiac surgery (%)	4312 (22.5)	36 (8.0)	4276 (22.8)		
Non‐postoperative care (%)	12 021 (62.7)	395 (87.4)	11 626 (62.1)		
No reaction to light (%)	163 (0.9)	112 (25.4)	51 (0.3)	< 0.001	1.3
Mechanical ventilation (%)	7651 (39.9)	356 (78.8)	7295 (38.9)	< 0.001	0.0
Systolic blood pressure, mmHg (median [IQR])	94.0 [80.0 to 110.0]	73.0 [56.5 to 96.0]	94.0 [80.0 to 110.0]	< 0.001	20.2
Base excess, mmol/L (median [IQR])	−2.0 [−5.0 to 1.1]	−7.0 [−14.9 to −0.2]	−2.0 [−5.0 to 1.1]	< 0.001	26.3
FiO_2_, % (median [IQR])	35.0 [21.0 to 40.0]	70.0 [40.0 to 100.0]	30.0 [21.0 to 40.0]	< 0.001	34.5
PaO_2_, mmHg (median [IQR])	100.5 [75.0 to 142.5]	80.2 [52.5 to 123.4]	102.0 [75.0 to 143.2]	< 0.001	47.8
FiO_2_/PaO_2_ mmHg (median [IQR])	339.7 [201.0 to 460.7]	138.2 [66.8 to 257.9]	347.5 [208.1 to 463.1]	< 0.001	48.5
Missing values (median [IQR])	1.0 [0.0 to 2.0]	0.0 [0.0 to 1.0]	1.0 [0.0 to 2.0]	< 0.001	0.0
EMR, % (median [IQR])	1.2 [0.5 to 2.2]	24.5 [7.3 to 79.0]	1.2 [0.5 to 2.0]	< 0.001	0.0
Deceased in ICU (%)	452 (2.4)	452 (100.0)	0 (0.0)	< 0.001	0.0

*Note:* Patients transported to another ICU were excluded. Numbers are No. (%) unless otherwise noted.

Abbreviations: ECC, extracorporeal circulation; EMR, estimated mortality rate according to original PIM3; FiO_2_, fraction of inspired oxygen; IQR, interquartile range; LOS, length of stay; Mechanical ventilation, mechanical ventilation within the first hour; PaO_2_, arterial partial pressure of oxygen.

**TABLE 2:** Treatments given in ICU to the admissions in all the ICU‐mortality cohort.

Variable	Overall	Deceased	Survived	*p*	Missing %
No.	19 183	452	18 731		
Invasive mechanical ventilation (%)	7719 (40.5)	390 (86.7)	7329 (39.3)	< 0.001	0.5
Non‐invasive mechanical ventilation (%)	1213 (6.4)	43 (9.6)	1170 (6.3)	0.007	0.5
ECMO (%)	162 (1.1)	77 (20.1)	85 (0.6)	< 0.001	26.0
CRRT (%)	234 (1.2)	80 (18.0)	154 (0.8)	< 0.001	1.3
Haemodialysis (%)	39 (0.2)	2 (0.4)	37 (0.2)	0.542	1.0

*Note:* Numbers are No. (%) unless otherwise noted.

Abbreviations: CRRT, continuous renal replacement therapy; ECMO, extracorporeal membrane oxygenation; IQR, interquartile range.

There were in total 21 410 admissions not discharged to another ICU which consequently should be included in the study. Of these, 2227 (10.4%) lacked a registered PIM3 score. Children with missing PIM3 scores were older than those with complete data, with a median age of 7 years (interquartile range [IQR] 2–13) compared with 2 years (IQR 0–9). The mortality rate among admissions without a PIM3 score was 14 per 1000 admissions compared to 24 per 1000 admissions in the study cohort.

The proportion of missing values for each PIM3 variable is presented in Table 1. ICU mortality, the primary outcome, had no missing data. Complete PIM3 data were available for 8969 (46.8%), while 4257 (22.2%) were missing one variable, 3784 (19.7%) two variables, 2115 (11.9%) three variables, and 58 (0.3%) four variables required for PIM3 calculation. The performance of PIM3 in cases with missing data in PICU and ICU is presented in Tables S1 and S2.

**FIGURE 2:**
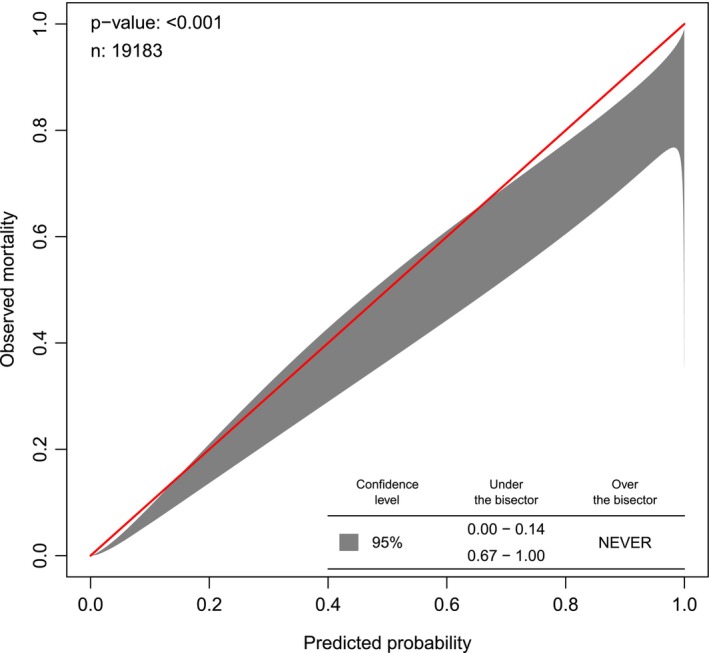
Predicted (original PIM3) and observed ICU‐mortality in the ICU‐mortality cohort plotted with calibration belt plot. (*n* = 19 183, *p* < 0.001). The calibration belt plot is adjusted for external validation. The *p*‐value suggests that the hypothesis of good calibration of the model is not met.

The overall SMR was 0.69 (95% CI 0.63–0.76) and the AUC‐ROC 0.93 (95% CI 0.92–0.95). The SMR was lower among admissions with an estimated mortality risk at or below the cohort median (1.2%) compared with those above the median. Specifically, the SMR was 0.27 (95% CI 0.15–0.44) in the low‐risk group and 0.74 (95% CI 0.67–0.81) in the high‐risk group. A calibration belt plot illustrating the variation in calibration between quantiles of predicted risk is shown in Figure 2. For transferred patients, the predicted mortality risk upon admission calculated by the PIM3 was higher in the first ICU (7.5%) than in the receiving PICU (6.6%).

### 3.1 Comparison Between PICU and ICU

For the comparison between PICU and ICU, we excluded 296 admissions to specialised ICUs including burns, neurology and cardiothoracic ICUs (Figure 1). After these exclusions, 12 001 admissions were to PICUs and 6886 to ICUs. Baseline characteristics of this cohort are presented in Table 3 and treatments received are summarised in Table 4.

**TABLE 3:** Baseline characteristics of admissions in the ICU/PICU cohorts.

Variable	Overall	ICU	PICU	*p*	Missing %
No.	18 887	6886	12 001		
Age (median [IQR])	2.0 [0.0 to 8.0]	6.0 [2.0 to 12.0]	1.0 [0.0 to 5.0]	< 0.001	0.0
Gender male (%)	10 548 (55.8)	3851 (55.9)	6697 (55.8)	0.884	0.0
ICU LOS, h (median [IQR])	23.1 [14.5 to 58.0]	17.8 [8.5 to 26.7]	31.2 [19.0 to 87.0]	< 0.001	0.0
Unplanned admission = No. (%)	6239 (33.0)	1102 (16.0)	5137 (42.8)	< 0.001	0.0
Very high risk admission (%)	852 (4.5)	270 (3.9)	582 (4.8)	0.003	0.0
High risk admission (%)	925 (4.9)	210 (3.0)	715 (6.0)	< 0.001	0.0
Low risk admission (%)	3386 (17.9)	1812 (26.3)	1574 (13.1)	< 0.001	0.0
Postoperative care (%)	7068 (37.4)	1345 (19.5)	5723 (47.7)	< 0.001	0.0
Cardiac surgery with ECC (%)	2455 (13.0)	9 (0.1)	2446 (20.4)		
Cardiac surgery without ECC (%)	392 (2.1)	2 (0.0)	390 (3.2)		
Non‐cardiac surgery (%)	4221 (22.3)	1334 (19.4)	2887 (24.1)		
Non‐postoperative care (%)	11 819 (62.6)	5541 (80.5)	6278 (52.3)		
No reaction to light (%)	154 (0.8)	77 (1.1)	77 (0.6)	< 0.001	1.3
Mechanical ventilation (%)	7487 (39.6)	1411 (20.5)	6076 (50.6)	< 0.001	0.0
Systolic blood pressure, mmHg (median [IQR])	93.0 [80.0 to 110.0]	100.0 [90.0 to 120.0]	90.0 [75.0 to 103.0]	< 0.001	20.3
Base excess, mmol/L (median [IQR])	−2.0 [−5.0 to 1.1]	−2.4 [−6.0 to 0.5]	−1.8 [−4.8 to 1.6]	< 0.001	26.3
FiO_2_, % (median [IQR])	35.0 [21.0 to 40.0]	25.0 [21.0 to 40.0]	40.0 [25.0 to 45.0]	< 0.001	34.5
PaO_2_, mmHg (median [IQR])	100.5 [75.0 to 142.5]	102.0 [81.8 to 136.5]	99.8 [73.5 to 145.5]	< 0.001	48.0
PaO_2_/FiO_2_, mmHg (median [IQR])	338.6 [201.5 to 457.5]	399.2 [255.0 to 507.1]	307.5 [187.5 to 432.9]	< 0.001	48.6
Missing values (median [IQR])	1.0 [0.0 to 2.0]	1.0 [0.0 to 2.0]	0.0 [0.0 to 2.0]	< 0.001	0.0
EMR (median [IQR])	1.2 [0.5 to 2.1]	1.2 [0.3 to 1.7]	1.2 [0.6 to 2.5]	< 0.001	0.0
Deceased in ICU (%)	400 (2.1)	103 (1.5)	297 (2.5)	< 0.001	0.0

*Note:* Numbers are No. (%) unless otherwise noted. Patients transported to another ICU were excluded.

Abbreviations: ECC, extracorporeal circulation; EMR, estimated mortality rate according to original PIM3; FiO_2_, fraction of inspired oxygen; ICU, intensive care unit; IQR, interquartile range; LOS, length of stay; Mechanical ventilation, mechanical ventilation within the first hour; PaO_2_, arterial partial pressure of oxygen; PICU, paediatric intensive care unit.

**TABLE 4:** Treatments of admissions in the ICU/PICU cohorts.

Variable	Overall	ICU	PICU	*p*	Missing %
No.	18 887	6886	12 001		
Invasive mechanical ventilation (%)	7554 (40.2)	1267 (18.7)	6287 (52.4)	< 0.001	0.5
Non‐invasive mechanical ventilation (%)	1207 (6.4)	316 (4.7)	891 (7.4)	< 0.001	0.5
ECMO (%)	110 (0.8)	0 (0.0)	110 (1.1)	< 0.001	25.2
CRRT (%)	205 (1.1)	38 (0.6)	167 (1.4)	< 0.001	0.1
Haemodialysis (%)	39 (0.2)	6 (0.1)	33 (0.3)	0.013	1.0

*Note:* Numbers are No. (%) unless otherwise noted.

Abbreviations: CRRT, continuous renal replacement therapy; ECMO, extracorporeal membrane oxygenation; ICU, intensive care unit; IQR, interquartile range; PICU, paediatric intensive care unit.

The SMR was 0.72 (95% CI 0.64–0.80) for PICUs and 0.52 (95% CI 0.42–0.63) for ICUs. Individual calibration belt plots for each unit type are shown in Figure S1. Missing data were more frequent among ICU admissions. The impact of missing values is detailed in Table S2.

### 3.2 Recalibration

For recalibration, the dataset including 19 183 admissions was randomly split into a development set comprising 15 289 admissions and a validation set comprising 3894 admissions (Tables S3a and S3b). In the validation set, the performance of the original model showed an AUC‐ROC of 0.94 (95% CI 0.91–0.96) and an overall SMR of 0.67 (95% CI 0.54–0.83). The SMR for PICU admissions was 0.67 (95% CI 0.51–0.87), and for ICU admissions, it was 0.57 (95% CI 0.35–0.88), as shown in Table S3b.

### 3.3 Simple Recalibration

A simple recalibration of the overall PIM3 score was performed to achieve perfect calibration (SMR = 1.00) in the development dataset, with the resulting formula −0.3581537 + 1.0819294 × original PIM3 score. In the validation dataset, this recalibration resulted in an SMR of 0.97 (95% CI 0.74–1.25) for PICU admissions and 0.86 (95% CI 0.52–1.32) for ICU admissions (Table S3b).

### 3.4 Full Recalibration

**FIGURE 3:**
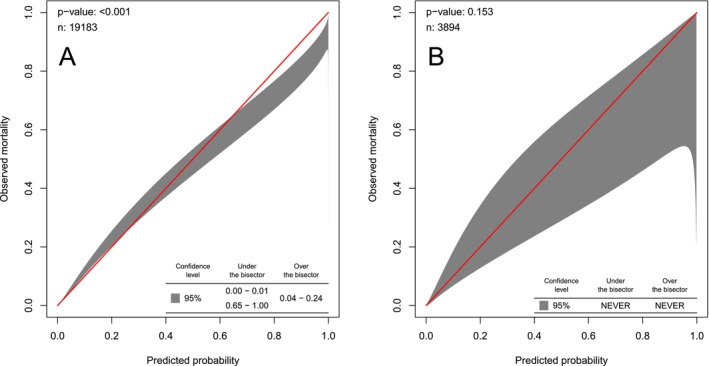
Calibration belt plot for the full recalibration of the model. (A) Internal plot of both development and validation (ICU‐mortality cohort, *n* = 19 183, *p* < 0.001). The *p*‐value suggests that the hypothesis of good calibration of the model is not met. (B) External plot of only validation cohort (*n* = 3894, *p* = 0.153). The *p*‐value suggests that the hypothesis of good calibration of the model is met. The curve has a sigmoid shape indicating that the calibration is not perfect in all spans of predicted mortality rate.

Full recalibration involved adjustment of all individual model coefficients. This yielded similar coefficients and had minimal impact on discrimination, with an AUC‐ROC of 0.94 (95% CI 0.91–0.96) in the validation dataset (Figure S3b). All predictors remained significant except for postoperative after cardiac surgery with and without extracorporeal circulation, which were non‐significant (*p* = 0.13 and *p* = 0.11, respectively). This approach reduced the difference in SMR between PICU and ICU to 0.92 (95% CI 0.70–1.20) and 0.96 (95% CI 0.59–1.49), respectively (Table S3b). The individual coefficients of the recalibrated model are presented in Table S4. Calibration belt plots of the recalibrated model are shown in Figure 3, while individual calibration plots for PICU and ICU are provided in Figure S2. Despite recalibration, the ICU cohort did not show a good fit in high‐risk admissions (Figure S2). Exclusion of 417 admissions who arrived at another ICU within 12 h of discharge improved the agreement between predicted and actual deaths for high‐risk admissions (Figure S3).

Elastic net shrinkage did not alter overall model performance but did affect the estimated coefficients, as detailed in Table S4. The coefficient most substantially affected by recalibration was elective admission.

## 4 Discussion

This study was the first assessment of the PIM3 severity of illness score in Scandinavia. The PIM3 showed excellent discrimination in our dataset, with an AUC‐ROC of 0.93, which exceeded results from previous validations [11]. However, the model overestimated the mortality risk indicated by an SMR of 0.69, and calibration belt plots revealed systematic bias. The SMR was lower for low‐risk admissions than for high‐risk admissions, which indicated insufficient risk adjustment, which suggested the need for recalibration [20]. It is important to note that the SMR calculated from mortality scores is likely to change over time, reflecting ever‐changing demographics in paediatric critical care settings.

We observed a higher SMR for admissions to specialised PICUs, with a more pronounced difference than previously reported for PIM2 in Sweden [4]. The discrepancy is unlikely to reflect inferior quality of care in PICUs compared to ICUs. Rather, it may reflect insufficient risk adjustment or inaccurate data reporting. Contributing factors could include a higher prevalence of high‐risk transfers to PICUs and severe conditions in specialised units, and under‐correction of high‐risk cases [23, 24]. Differences in data collection practices between Sweden and the context in which the original model was developed may also have affected this result. Patients transferred from one ICU to another are likely to show physiological improvement due to initial treatment and must be stable enough for transfer. Our dataset supports this, showing that the predicted mortality risk upon admission was higher in the first ICU than in the receiving PICU for transferred patients.

Admissions with more than one missing physiological variable showed a tendency toward a higher SMR compared with admissions with complete data, while model discrimination was highest in admissions without missing data. Overall, the limited impact of a small number of missing physiological variables supports the established PIM3 approach to handling missing data.

A higher proportion of missing values leads to an increased SMR because missing values are treated as normal, which lowers the estimated mortality rate if the actual missing values were abnormal. The proportion of missing data was higher in ICUs compared to PICUs, which may contribute to an artificially elevated SMR in the ICU cohort. One probable explanation for the higher proportion of missing data in ICUs is that less severely ill patients were less likely to undergo certain tests based on the clinical judgement of the physician, such as arterial blood gas analysis [10]. However, this does not appear to adversely affect comparisons between ICUs and PICUs, as the SMR was lower in ICUs. The higher SMR in the PICU may be explained by the differences in how values are reported. In the PIM3, the first measured value in the first hour is recorded, whereas in SAPS3, the model used for adult severity scoring in Sweden, the worst value within 1 h of admission is recorded. This may result in the worst value being recorded instead of the first value. In addition, the less frequent use of PIM3 in the Swedish ICUs may impact the accuracy of risk assessment. Overall, the model seems to perform more reliably in the PICU population than in the ICU population (Figures S1 and S2).

Recalibration of individual coefficients of the model resulted in a small improvement in performance compared to an overall recalibration, with the recalibrated coefficients remaining closely aligned to the original values. Nevertheless, recalibration did not result in a good fit in high‐risk admissions in the ICU cohort. However, recalibration reduced the difference in SMRs of the PICU and ICU cohorts. Recalibration of the model resulted in improved applicability of the model to the study cohort and reduced differences in SMR in PICU and ICU. Despite the relatively large sample size, confidence intervals for SMRs were wide because mortality in paediatric intensive care is low, which should be considered when comparing SMRs across paediatric intensive care units.

The lower than predicted mortality among high‐risk admissions may reflect inaccurate recording of discharge destination. PIM3 predicts ultimate ICU mortality, so transfers to another ICU should be excluded. We found that excluding admissions of patients that arrived at another PICU or ICU within 12 h of discharge improved calibration in the high‐risk group. This implies that ultimate ICU mortality is not always reliable in our setting.

### 4.1 Strengths and Limitations

This study was the first to evaluate the performance of the PIM3 score in a Scandinavian context, providing new regional insights and expanding the external validation of the PIM3 model. The dataset comprises a large national cohort, covering most intensive care of children in Sweden over more than 6 years. To the best of our knowledge, this is the only validation of the PIM3 model in Sweden to date. In addition, ICU mortality data were complete and the dataset was recent. The main limitation is that while data submitted to the Swedish Intensive Care Registry are validated to ensure they fall within pre‐specified limits before inclusion in the database, no comprehensive validation against individual patient records has been performed to date. Although data from all ICUs in Sweden were included, 21 of the 84 ICUs did not record PIM3. These were predominantly smaller ICUs, resulting in 10.4% of admissions lacking PIM3 registration. As the age and mortality of the admissions differ, they are likely to manage a different patient profile compared with larger centres. This incomplete coverage may have influenced the case‐mix of the study population.

The COVID‐19 pandemic, which spans part of the study period, may have influenced case‐mix and illness severity through altered patterns of respiratory illness, delayed presentations and the emergence of a novel hyperinflammatory syndrome in children. During the pandemic, the number of intensive care admissions of children decreased in Sweden. This should be considered when interpreting findings across the study period.

## 5 Conclusion

This study demonstrated that PIM3 had excellent discrimination in the Swedish paediatric population. The overestimation of mortality by the PIM3 in this cohort highlighted the need for recalibration. Recalibration corrected this bias and attenuated outcome differences between PICU and ICU settings. However, the recalibrated model performed less well in high‐risk admissions. This may be explained by incorrect registration of discharge destination, and ultimate ICU mortality does not seem to be a perfectly reliable endpoint in our setting.

## Author Contributions

**Lars Engerström:** formal analysis, visualization, conceptualization, methodology, data curation, writing – review and editing, writing – original draft, project administration, investigation. **Staffan Eksborg:** methodology, writing – review and editing, conceptualization. **Shanay Daham:** writing – original draft, writing – review and editing. **Jonas Berner:** writing – original draft, writing – review and editing, project administration, conceptualization, methodology. **Tova Hannegård Hamrin:** writing – review and editing, methodology, conceptualization.

## Funding

The authors have nothing to report.

## Disclosure

The authors disclose that generative AI or other AI‐assisted technologies were not used in the writing process.

## Conflicts of Interest

The authors declare no conflicts of interest.

## Supporting information


**Figure S1:** Calibration belt plots for the original PIM3 model in (A) the general ICU population (*n* = 6886; *p* < 0.001) and (B) the paediatric ICU population (*n* = 12 001; *p* < 0.001). Both the development and validation cohorts are included. The calibration belt plots are adjusted for external validation. The *p*‐values suggests that the hypothesis of good calibration of the model is not met. ICU, intensive care unit; PICU, paediatric intensive care unit.
**Figure S2:** Calibration belt plots for the recalibrated PIM3 model in (A) the general ICU population (*n* = 6886; *p* < 0.001) and (B) the paediatric ICU population (*n* = 12 001; *p* = 0.013). Both the development and validation cohorts are included. The calibration belt plots are adjusted for internal validation. The *p*‐values suggests that the hypothesis of good calibration of the model is not met. ICU, intensive care unit; PICU, paediatric intensive care unit.
**Figure S3:** Calibration belt plots for the recalibrated PIM3 model where patients admitted to another ICU within 12 h of discharge are removed to exclude those discharged to another ICU but registered with an incorrect discharge destination. (A) the general ICU population (*n* = 6626; *p* < 0.001) and (B) the paediatric ICU population (*n* = 11 849; *p* < 0.001). Both the development and validation cohorts are included. The calibration belt plots are adjusted for internal validation. The *p*‐values suggests that the hypothesis of good calibration of the model is not met. ICU, intensive care unit; PICU, paediatric intensive care unit.
**Table S1:** Performance of the PIM3 model with different number of missing variables.
**Table S2:** Number of missing variables and resulting SMR by ICU type.
**Table S3a:** Performance of the models, development.
**Table S3b:** Performance of the models, validation.
**Table S4:** Coefficients of the recalibrated models.

## Data Availability

The data that support the findings of this study are available from Swedish intensive care registry. Restrictions apply to the availability of these data, which were used under license for this study. Data are available from http://www.icuregswe.org with the permission of Swedish intensive care registry.
