# Supplementary material for: Paediatric Index of Mortality 3 in Sweden: Validation and Recalibration to Improve Mortality Prediction in Paediatric Intensive Care
Source: Acta Paediatr. 2026 May 20;115(9):2008–17. doi: 10.1111/apa.70603 (PMC13460836; doi:10.1111/apa.70603)
Supplement: Supplementary file 1 [file APA-115-2008-s001.pdf]

Supplementary material

Table S1.

No of missing values	No	Deceased	EMR	AUC_{ROC}	SMR
All dataset	18887	400	614	0.93 (0.91 - 0.93)	0.65
0	8797	255	390	0.94 (0.93 - 0.94)	0.65
1	4214	77	125	0.90 (0.86 - 0.90)	0.62
2	3722	48	70	0.92 (0.88 - 0.92)	0.68
3	2098	20	28	0.87 (0.81 - 0.87)	0.71
4	56	0	0		0

Performance of the PIM3 model with different number of missing variables. EMR = estimated mortality risk. AUC_{ROC} = area under the receiver operating characteristic curve, presented with 95% confidence intervals. SMR = standardised mortality ratio, calculated using the original formula. ICU = intensive care unit. PICU = paediatric intensive care unit. Results are shown for the combined ICU and PICU cohorts, not the full ICU-mortality cohort.

Table S2.

	All			PICU			ICU		
No of missing values	No.	Deceased	SMR	No.	Deceased	SMR	No.	Deceased	SMR
All dataset	18887	400	0.65	12001	297	0.72	6886	103	0.52
0	8797	255	0.65	6270	197	0.69	2527	58	0.55
1	4214	77	0.62	2589	54	0.68	1625	23	0.51
2	3722	48	0.68	2021	35	0.95	1701	13	0.39
3	2098	20	0.71	1109	11	0.8	989	9	0.62
4	56	0	0	12	0	0	44	0	0

Number of missing variables and resulting SMR by ICU type.
SMR = standardised mortality ratio, calculated using the original formula. ICU = intensive care unit. PICU = paediatric intensive care unit. Results are presented separately for the PICU and ICU cohorts. SMR = standardised mortality ratio, calculated using the original formula.

Table S3a. Performance of the models, development

Model	Development n=15 289<br>AUC$_{ROC}$	SMR	HLCp	PICU n=9 579<br>SMR	ICU n=5 479<br>SMR
Original	0.933 (0.919 - 0.946)	0.70 (0.63 - 0.77)	<0.001	0.73 (0.64 - 0.83)	0.50 (0.40 - 0.63)
Overall recalibration	0.933 (0.919 - 0.946)	1.00 (0.90 - 1.11)	0.13	1.08 (0.95 - 1.23)	0.71 (0.57 - 0.88)
Full recalibration	0.936 (0.923 - 0.950)	1.00 (0.90 - 1.11)	0.35	1.05 (0.92 - 1.19)	0.77 (0.61 - 0.95)

The full development cohort included admissions to PICU and ICU, as well as to specialised neuro-, cardiothoracic-, ECMO-, and burns-ICUs. Overall recalibration refers to adjustment of the PIM3 model using an overall recalibration factor (–0.3581537 + 1.0819294 × original PIM3 score) to fit the development cohort. Full recalibration refers to re-estimation of all individual model coefficients to optimise fit within the development cohort. ICU = intensive care unit. PICU = paediatric intensive care unit. SMR = standardised mortality ratio. HLCp= Hosmer-Lemeshow test p-value.

Table S3b. Performance of the models, validation.

Model	Validation n=3 894<br>AUC$_{ROC}$	SMR	HLCp	PICU n=2 422<br>SMR	ICU n=1 407<br>SMR
Original	0.935 (0.909 - 0.961)	0.67 (0.54 - 0.83)	0.005	0.67 (0.51 - 0.87)	0.57 (0.35 - 0.88)
Overall recalibration	0.935 (0.909 - 0.961)	0.97 (0.78 - 1.20)	0.87	0.97 (0.74 - 1.25)	0.86 (0.52 - 1.32)
Full recalibration	0.937 (0.912 - 0.962)	0.96 (0.77 - 1.19)	0.31	0.92 (0.70 - 1.20)	0.96 (0.59 - 1.49)

The full development cohort included admissions to PICU and ICU, as well as to specialised neuro-, cardiothoracic-, ECMO-, and burns-ICUs. Overall recalibration refers to adjustment of the PIM3 model using an overall recalibration factor (–0.3581537 + 1.0819294 × original PIM3 score) to fit the development cohort. Full recalibration refers to re-estimation of all individual model coefficients to optimise fit within the development cohort. ICU = intensive care unit. PICU = paediatric intensive care unit. SMR = standardised mortality ratio. HLCp= Hosmer-Lemeshow test p-value.

Table S4. Coefficients of the recalibrated models.

Variable	Original	Full recalibration	Elastic net	Ratio
Constant	-1.7928	-2.7706	-2.8647	1.60
Systolic blood pressure	-0.0431	-0.0393	-0.0369	0.86
Systolic blood pressure squared	0.1716	0.1327	0.1187	0.69
No reaction to light	3.8233	3.2137	3.2196	0.84
FiO_2/PaO_2 (mmHg)	0.4214	0.7637	0.7667	1.82
Mechanical Ventilation	0.9763	0.9938	0.9825	1.01
Base excess	0.0671	0.0672	0.0671	1.00
Elective admission	-0.5378	-1.2442	-1.2385	2.30
Postoperative non-cardiac	-1.5164	-0.7074	-0.6941	0.46
Postoperative cardiac with ECC	-1.2246	-0.484	-0.4701	0.38
Postoperative cardiac without ECC	-0.8762	-1.1571	-1.0999	1.26
Very High Risk	1.6225	2.2311	2.2352	1.38
High Risk	1.0725	1.4453	1.4404	1.34
Low Risk	-2.1766	-2.9472	-2.7859	1.28

Original refers to the original PIM3 model. Full recalibration denotes the complete recalibration of all individual model coefficients. Elastic net indicates the application of coefficient shrinkage using elastic net regularisation to reduce overfitting. Ratio represents the ratio between the coefficients obtained using elastic net and the original coefficients, illustrating the magnitude of change.

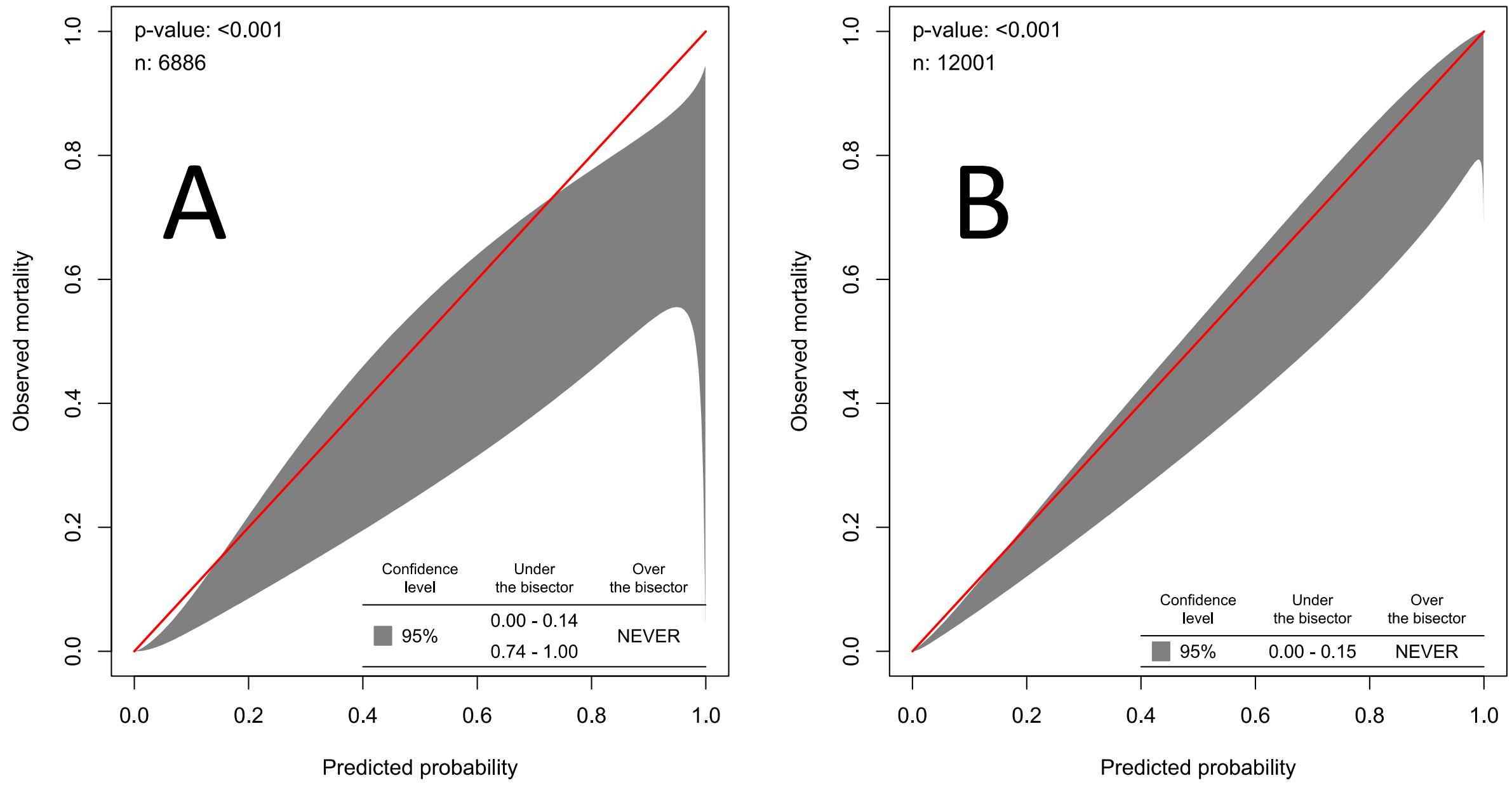

Figure S1. Calibration belt plots for the original PIM3 model in (A) the general ICU population (n = 6 886; p < 0.001) and (B) the paediatric ICU population (n = 12 001; p < 0.001). Both the development and validation cohorts are included. The calibration belt plots are adjusted for external validation. The p-values suggests that the hypothesis of good calibration of the model is not met. ICU = intensive care unit. PICU = paediatric intensive care unit.

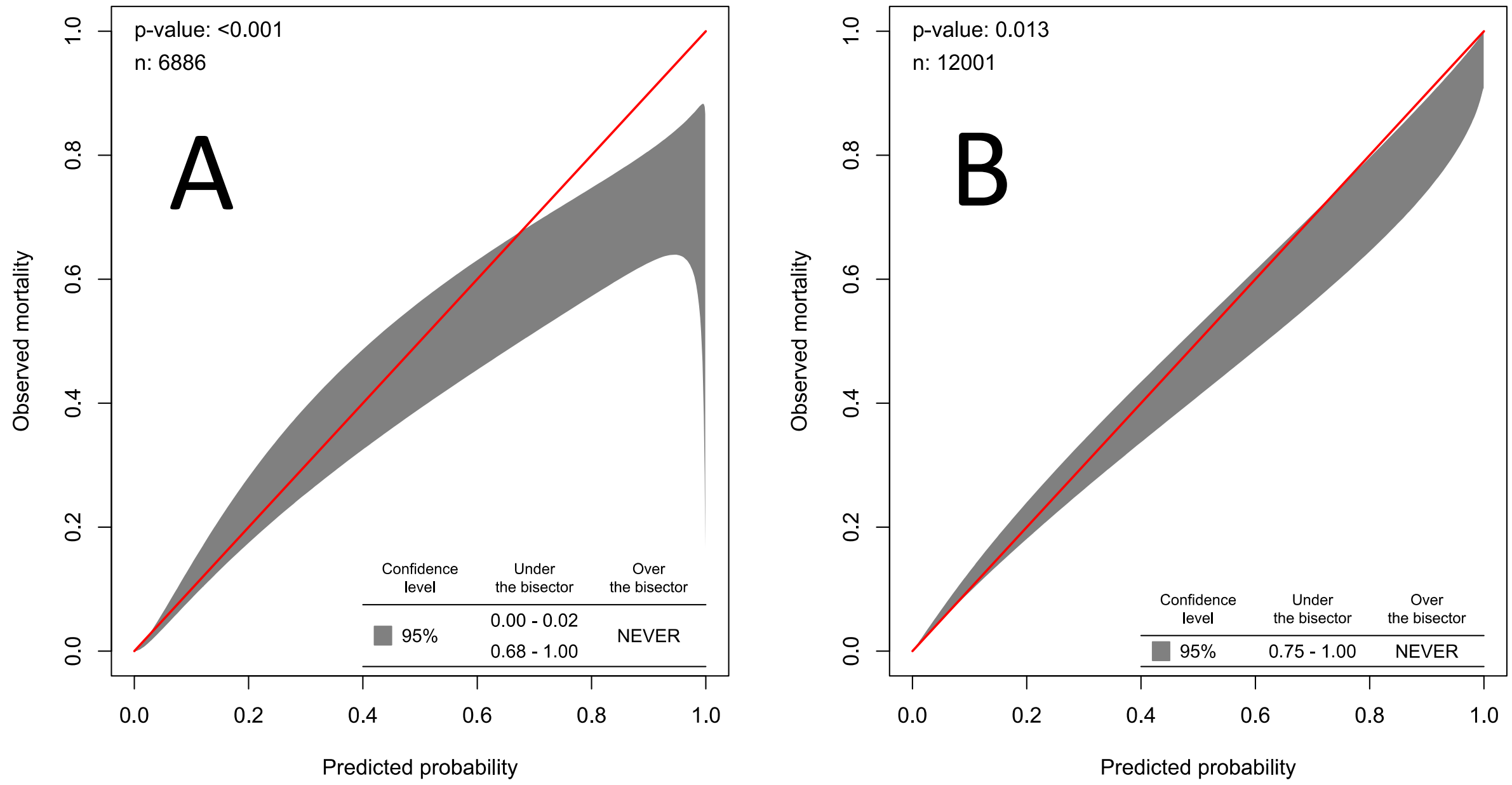

Figure S2. Calibration belt plots for the recalibrated PIM3 model in (A) the general ICU population (n = 6 886; p < 0.001) and (B) the paediatric ICU population (n = 12 001; p = 0.013). Both the development and validation cohorts are included. The calibration belt plots are adjusted for internal validation. The p-values suggests that the hypothesis of good calibration of the model is not met. ICU = intensive care unit. PICU = paediatric intensive care unit.

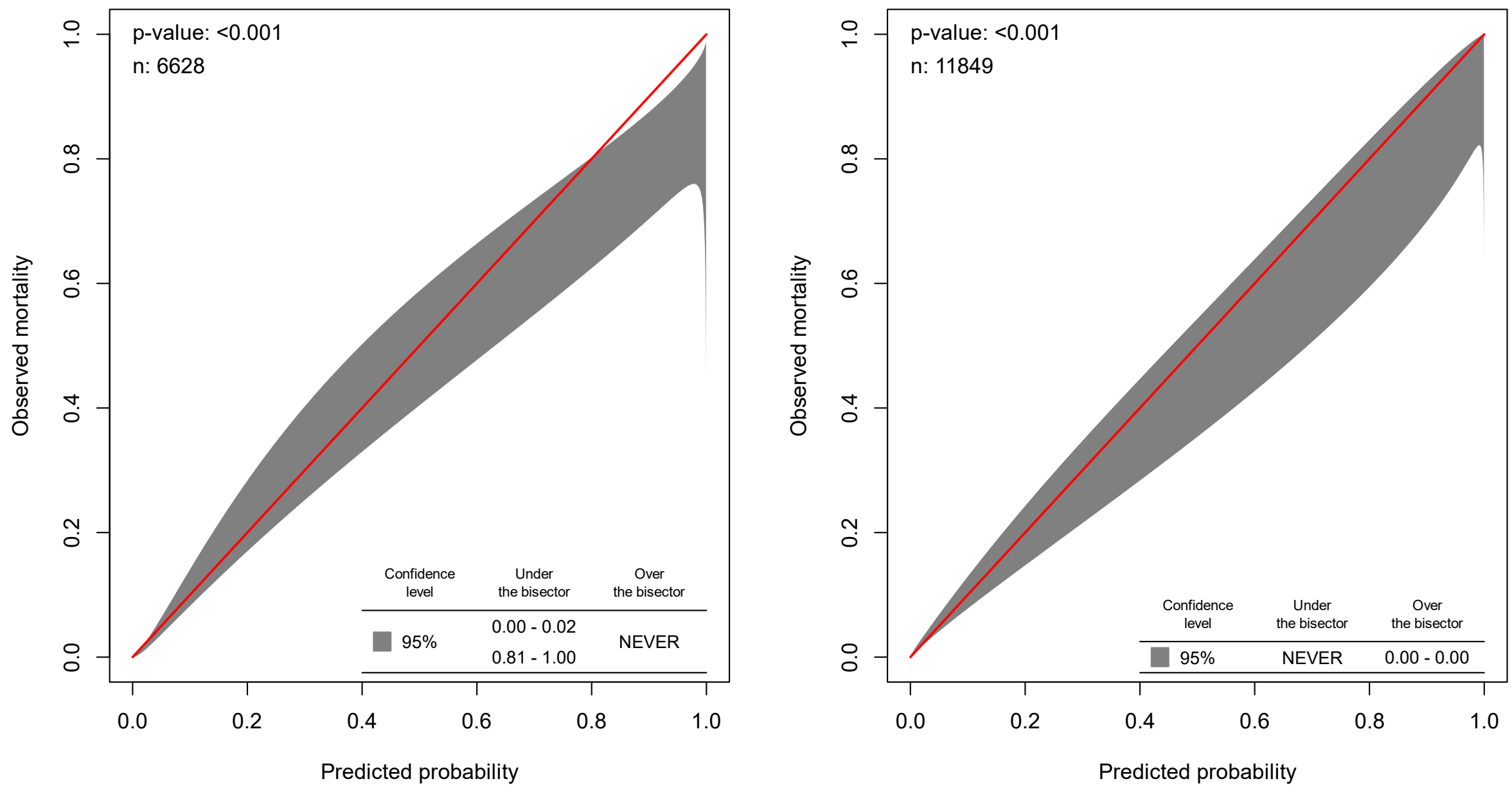

Figure S3. Calibration belt plots for the recalibrated PIM3 model where patients admitted to another ICU within 12 hours of discharge are removed to exclude those discharged to another ICU but registered with an incorrect discharge destination. (A) the general ICU population (n = 6 626; p < 0.001) and (B) the paediatric ICU population (n = 11 849; p <0.001). Both the development and validation cohorts are included. The calibration belt plots are adjusted for internal validation. The p-values suggests that the hypothesis of good calibration of the model is not met. ICU = intensive care unit. PICU = paediatric intensive care unit.
